# Involvement of the E2-like enzyme Atg3 in fungal development and virulence of *Botryosphaeria dothidea*

**DOI:** 10.3389/fpls.2025.1590359

**Published:** 2025-08-15

**Authors:** Wenjiao Han, Yang Han, Yanru Ma, Guolei Yu, Xushi Jiang, Qian Yang, Na Liu, Caixia Wang, Baohua Li, Sen Lian, Weichao Ren

**Affiliations:** Shandong Engineering Research Center for Environment-Friendly Agricultural Pest Management, College of Plant Health and Medicine, Qingdao Agricultural University, Qingdao, China

**Keywords:** *Botryosphaeria dothidea*, autophagy, Atg3, development, virulence

## Abstract

Autophagy is a fundamental cellular degradation and recycling system that is crucial for maintaining cellular homeostasis, responding to stress, and ensuring the proper functioning of cells. To date, the biological functions of autophagy in the plant-pathogenic fungus *Botryosphaeria dothidea* remains largely unknown. In this study, we identified and characterized the E2-like enzyme Atg3 in *B. dothidea*. The autophagic process was blocked in the *BdATG3* deletion mutant Δ*BdAtg3*, and the Δ*BdAtg3* mutant showed serious defects in mycelial growth, conidiation, perithecium formation and virulence. In addition, the Δ*BdAtg3* mutant exhibited an increased number of nuclei in mycelial compartment. All of the phenotypic changes of the Δ*BdAtg3* mutant were restored by gene complementation. These results indicate that the E2-like enzyme Atg3 plays an important role in various developmental processes and pathogenesis of *B. dothidea*, which provides a potential target for developing novel fungicides.

## 1 Introduction

Autophagy is a fundamental cellular process that involves the degradation and recycling of unnecessary or dysfunctional components within the cell (Ohsumi, 2012; Parzych and Klionsky, 2014). This catabolic mechanism allows cells to maintain homeostasis by eliminating damaged organelles and proteins, thereby ensuring efficient cellular function and survival (Eskelinen, 2019; Mizushima, 2007). Atg3 is essential for the lipidation of Atg8, a key step in autophagosome formation (Martens and Fracchiolla, 2020). It acts as an E2-like enzyme, working in concert with Atg7 and the Atg12-Atg5 complex to ensure proper conjugation of Atg8 to phosphatidylethanolamine (PE) (Fang et al., 2021; Mizushima, 2020). This process is vital for the formation, maturation, and function of autophagosomes, ultimately enabling the degradation of cellular components and maintenance of cellular homeostasis (Zhao and Zhang, 2018). Several studies have highlighted the crucial role of Atg3 in fungal development and virulence in filamentous fungi, including *Magnaporthe oryzae*, *Botrytis cinerea*, and *Fusarium graminearum* (Lv et al., 2017; Pollack et al., 2009; Ren et al., 2018b; Yin et al., 2019), but its role in *Botryosphaeria dothidea* remains unknown.

Botryosphaeria canker and apple ring rot, caused by the fungal pathogen *Botryosphaeria dothidea*, is a destructive disease affecting apple trees worldwide (Dong et al., 2021; Liu et al., 2022a). This pathogen is a member of the family Botryosphaeriaceae and is known for its ability to infect a wide range of woody plants, including apple, pear, and grapevine (Marsberg et al., 2017). This disease is particularly problematic in regions with warm and humid climates that causes damage during both the growing season and the storage period making it a significant concern for apple growers worldwide, leading to substantial economic losses (Ren et al., 2023; Sun et al., 2023). Currently, the absence of disease-resistant apple varieties makes chemical control the most effective strategy for managing apple ring rot (Zhu et al., 2024). However, the long-term and extensive use of fungicides has led to the emergence of *B. dothidea* strains resistant to some chemical fungicides, such as benzimidazoles (Wang et al., 2022). Therefore, there is an urgent need to develop more efficient fungicides. Investigating the molecular mechanisms governing vegetative differentiation, pathogenicity, and stress responses in *B. dothidea* is crucial for identifying potential targets for novel fungicide development, paving the way for more sustainable and effective disease management. In this study, we identified and characterized the E2-like enzyme BdAtg3 in *B. dothidea*, and the results provides a theoretical basis for the development of novel target-based fungicide for the control of apple ring rot disease.

## 2 Materials and methods

### 2.1 Fungal strains and culture conditions

The wild-type (WT) strain LW03 (LXS030101) of *B. dothidea* was used as parental strain for gene knockout. All of the *B. dothidea* strains were cultured on potato dextrose agar (PDA) (200 g potato, 20 g glucose, 10 g agar and 1 L water), minimal medium (MM) (0.5 g KCl, 2 g NaNO_3_, 1 g KH_2_PO_4_, 0.5 g MgSO4·7H_2_O, 0.01 g FeSO4·7H_2_O, 30 g sucrose, 200 μL trace element, 15 g agar, 1 L water, pH 6.9), and complete medium (CM) [1% glucose, 0.2% peptone, 0.1% yeast extract, 0.1% casamino acids, nitrate salts (6 g NaNO_3_, 0.52 g KCl, 0.52 g MgSO_4_·7H_2_O, 1.52 g KH_2_PO_4)_, trace elements, 0.01% vitamins (biotin, pyridoxine, thiamine, riboflavin, p-aminobenzoic acid, and nicotinic acid), 1 L water, pH 6.5) at 25°C for mycelial growth. Young fruits of ‘Fuji’ apple were used for conidial production, and one-year-old branches of ‘Fuji’ apple were used for perithecial formation.

### 2.2 Gene deletion and complementation

The gene knockout constructs of *BdATG3* were generated using a double-joint PCR method (Yu et al., 2004), and introduced into WT strain thorough PEG-mediated protoplast transformation. *BdATG3* was replaced by hygromycin resistant gene (*HPH*) based on homologous recombination strategy (**Supplementary Figure S1A**). The putative deletion mutants with hygromycin resistance (100 µg/mL) were identified by PCR amplification analysis. For complementation, the fragment containing the full-length of *BdATG3* and native promoter, which was predicted through the promoter prediction website (https://fruitfly.org/seq_tools/promoter.html), was inserted into PYF11 vector (Liu et al., 2022a) and transformed into the *BdATG3* deletion mutant.

### 2.3 Protein manipulation and Western blotting

The GFP-BdAtg8 fusion construct was generated as described previously (Liu et al., 2022a). For protein extraction, the fresh mycelia of *B. dothidea* were ground into fine powder in liquid nitrogen, and resuspended in protein extraction buffer with protease inhibitor cocktail (Sangon, Shanghai, China), and centrifuged at 12000 rpm for 10 min at 4°C. The supernatant was mixed with an equal volume of protein loading buffer and boiled for 5 minutes to denature the proteins, which were loaded onto a 12.5% SDS-PAGE and transferred to a PVDF (polyvinylidene fluoride) membrane that was pre-activated by soaking in methanol. The monoclonal anti-GFP antibody (ab32146, Abcam, Cambridge, MA, USA) and monoclonal anti-glyceraldehyde-3-phosphate dehydrogenase (GAPDH) antibody (EM1101, Hangzhou Huaan Biotechnology Co., Ltd., Hangzhou, China) were used for immunoblot analyses.

### 2.4 Determination of asexual and sexual reproduction

For conidial production, young apple fruits were inoculated with each strain, and exposed to UV irradiation (365 nm wavelength) in 25°C to induce conidiation. After 14 days, morphological development, including the formation of fruiting bodies and production of conidia or microspores, was monitored using light microscopy. For sexual reproduction, the infected apple branches were maintained in a moist chamber with periodic water spraying at ambient temperature (16-28°C). The development of perithecia was examined after 30 days of incubation. Both experiments were repeated 3 times.

### 2.5 Pathogenicity tests

The pathogenicity of *B. dothidea* was evaluated through artificial inoculation assays using detached fruits, branches, and leaves of ‘Fuji’ apple. Prior to inoculation, the plant tissues were surface-sterilized and wounded using a sterile needle. Mycelial plugs (5 mm diameter) from the actively growing margins of 7-day-old cultures were transferred to the wound sites. Control samples received sterile PDA plugs without fungal inoculation. All inoculated samples were maintained in a controlled environment chamber at 25°C with 95% relative humidity. Disease progression was monitored daily, and lesion diameters were measured at 7 days post-inoculation (dpi). The experiment was conducted in 3 independent biological replicates, with 10 technical replicates per treatment in each experiment.

### 2.6 Statistical analysis

Statistical analysis was performed using SPSS 20 software (IBM, Armonk, NY, USA). Multiple comparisons were conducted using Fisher’s Least Significant Difference (LSD) test and Duncan’s new multiple range test. Error bars in the figures indicate the standard deviation (SD) derived from triplicate independent experiments. Mean values labeled with the same lowercase letter above the bars indicate no statistically significant difference at the 0.05 probability level according to the *post hoc* tests.

## 3 Results

### 3.1 Identification of BdAtg3 in *B. dothidea*

**Figure 1:**
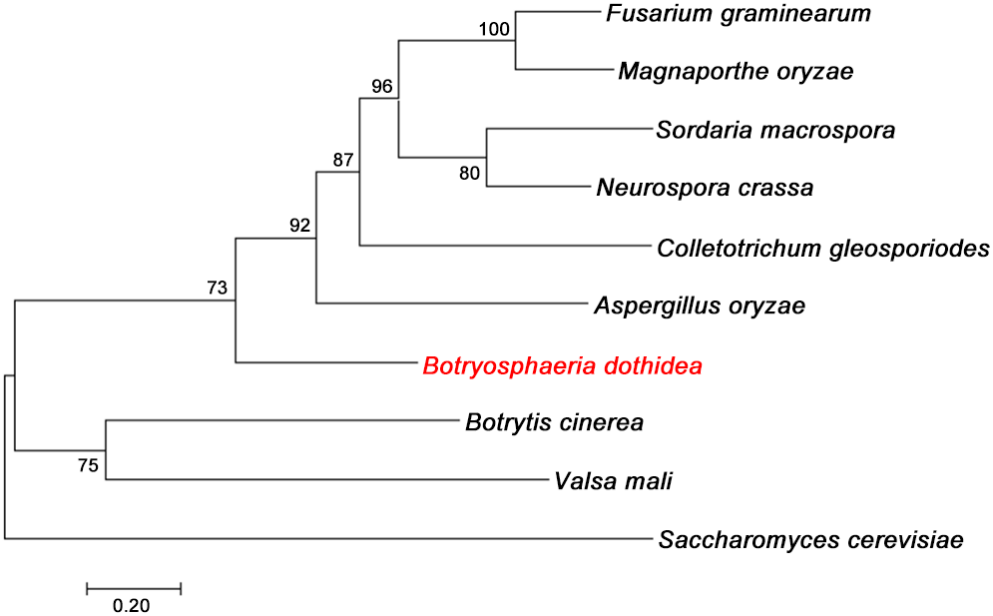
Evolutionary analysis of BdAtg3. Phylogenetic tree of BdAtg3 with other orthologs from *Fusarium graminearum* (XP_011319806), *Magnaporthe oryzae* (XP_003720747), *Sordaria macrospora* (XP_003347100), *Neurospora crassa* (XP_062690851), *Colletotrichum gleosporiodes* (XP_045261435), *Aspergillus oryzae* (XP_001823534), *Botrytis cinerea* (XP_024550449), *Valsa mali* (KUI73004), and *Saccharomyces cerevisiae* (CAI4696490).

To identify and characterize the autophagy protein BdAtg3 in *B. dothidea*, the well-characterized Atg3 protein from *Saccharomyces cerevisiae* was used as a query for BLASTp search against the *B. dothidea* genome database (https://www.ncbi.nlm.nih.gov/biosample/SAMN13735646). The putative BdAtg3 protein consists of 357 amino acids that with 35.2% identity to Atg3 ortholog of *S. cerevisiae*, and contains the typical ubiquitin-like protein (UBL) domain. Phylogenetic analysis by neighbor-joining method revealed that BdAtg3 cluster closely with the orthologs from *Fusarium graminearum*, *Magnaporthe oryzae*, *Sordaria macrospora*, *Neurospora crassa*, *Colletotrichum gleosporiodes*, *Aspergillus oryzae* (**Figure 1**), suggesting the evolutionary conservation among filamentous fungi.

To investigate the biological function of BdAtg3 in *B. dothidea*, we employed a homologous recombination strategy to generate *BdATG3* deletion mutants (**Supplementary Figure S1A**). The putative deletion mutants were screened by hygromycin resistance (100 µg/mL), and further identified by PCR amplification analysis using diagnostic primer pair P7/P8 (**Supplementary Table S1**), the amplicons of wild-type (WT) strain and Δ*BdAtg3* mutants were 1579 bp and 1786 bp, respectively (**Supplementary Figure S1B**), indicating that *BdATG3* was successfully replaced by *HPH* cassette.

### 3.2 BdAtg3 is essential for autophagy

**Figure 2:**
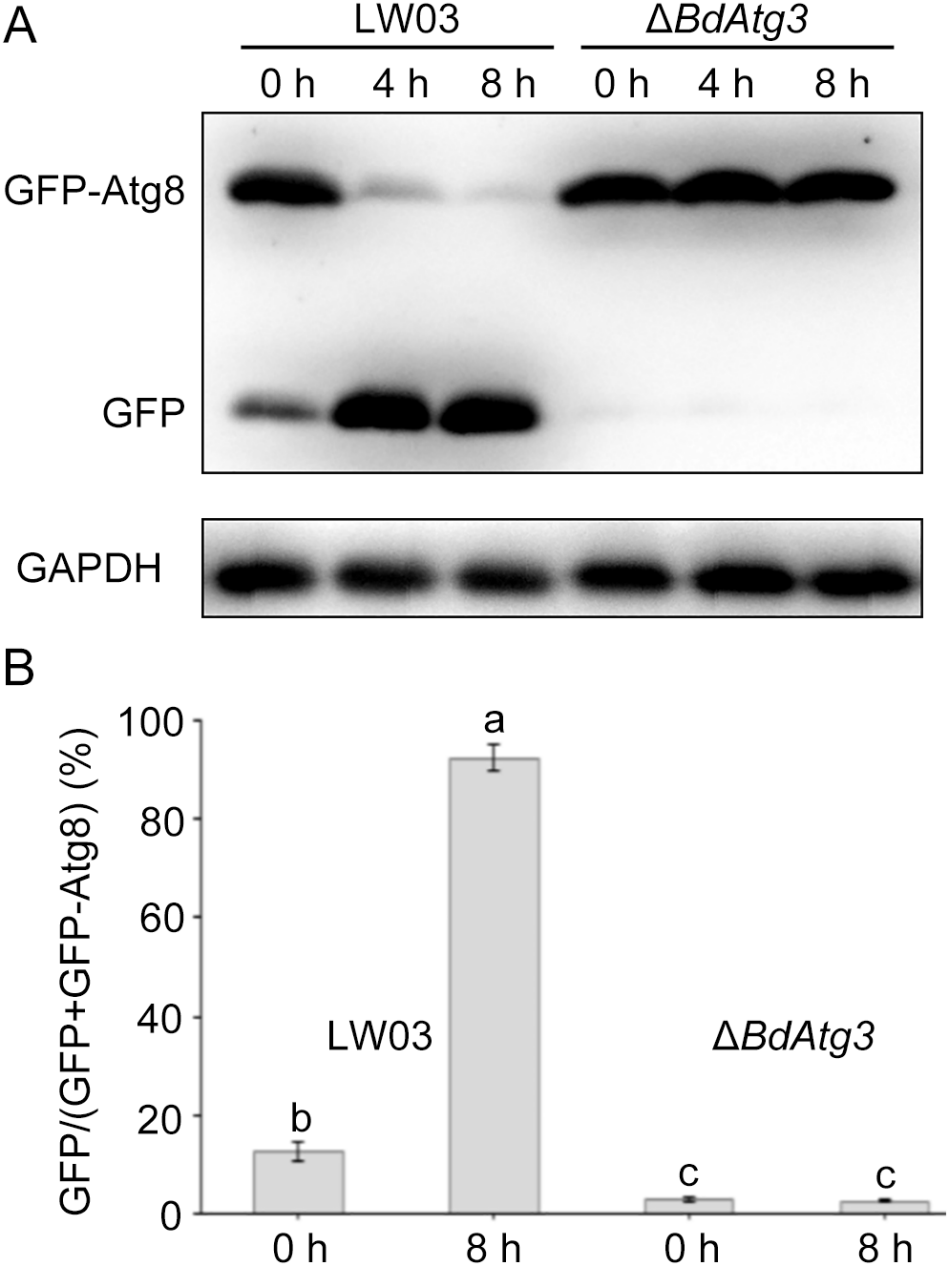
The necessity of BdAtg3 for autophagy. **(A)** Western blot analysis of the proteolysis of GFP-BdAtg8 in the wild-type strain LW03 and *BdATG3* deletion mutant Δ*BdAtg3* after 0 h, 4 h, and 8 h of nitrogen starvation. GAPDH served as the internal control. **(B)** The percentage of GFP on the total of GFP and GFP-BdAtg8. Error bars indicate standard deviation from three independent experiments, and values on the bars followed by the same letter are not significantly different at *P* = 0.05.

The GFP-Atg8 fusion protein has been widely established as a reliable molecular marker for monitoring autophagic flux in eukaryotic cells (Nair et al., 2011). To investigate the functional role of BdAtg3 in autophagy regulation, we employed a GFP-BdAtg8 reporter system to compare autophagic activity between the wild-type strain LW03 and the Δ*BdAtg3* mutant. The immunoblot analysis of GFP-BdAtg8 processing demonstrated a significant reduction in full-length GFP-BdAtg8 protein levels in LW03 following nitrogen starvation, while Δ*BdAtg3* maintained stable GFP-BdAtg8 protein levels (**Figures 2A, B**). These results indicate that BdAtg3 is indispensable for autophagy induction and progression in *B. dothidea*.

### 3.3 BdAtg3 is involved in mycelial growth

**Figure 3:**
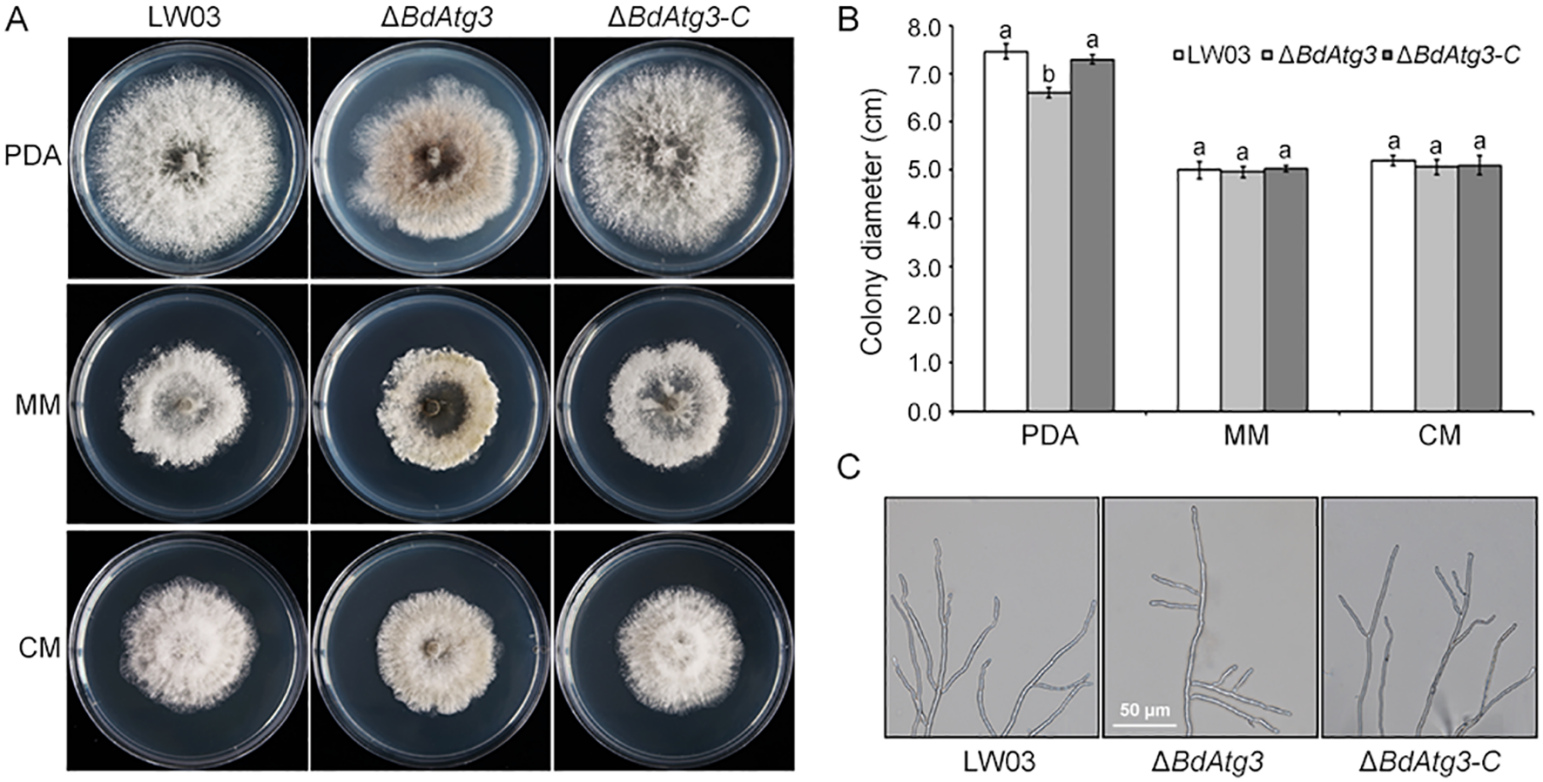
BgAtg3 is involved in mycelial growth. **(A)** Mycelial growth of the wild-type strain LW03, *BdATG3* deletion mutant Δ*BdAtg3* and complemented strain Δ*BdAtg3-C* on PDA, MM, and CM media at 25°C for 3 days. **(B)** Mycelial growth rate of each strain in PDA, MM, and CM media. Values on the bars followed by the same letter are not significantly different at *P* = 0.05. **(C)** Morphology of the aerial hyphae tips in each strain.

To investigate the functional role of BdAtg3 in mycelial growth, *B. dothidea* strains were grown on PDA and MM media. The Δ*BdAtg3* mutant exhibited a ~10% reduction in radial growth rate on PDA plates and produced fewer aerial hyphae compared with the wild-type strain LW03 and complemented strain Δ*BdAtg3-C* after 3 days of incubation at 25°C (**Figures 3A, B**). Morphological observation thorough microscope (Olympus CX43, Tokyo, Japan) showed that Δ*BdAtg3* displayed abnormal hyphal tip growth patterns, characterized by excessive apical branching and hyphal twisting after cultured on PDA 2 days (**Figure 3C**). These results suggest that BdAtg3 is involved in normal mycelial growth and cellular morphogenesis in *B. dothidea*.

### 3.4 BdAtg3 is essential for sexual and asexual reproduction

**Figure 4:**
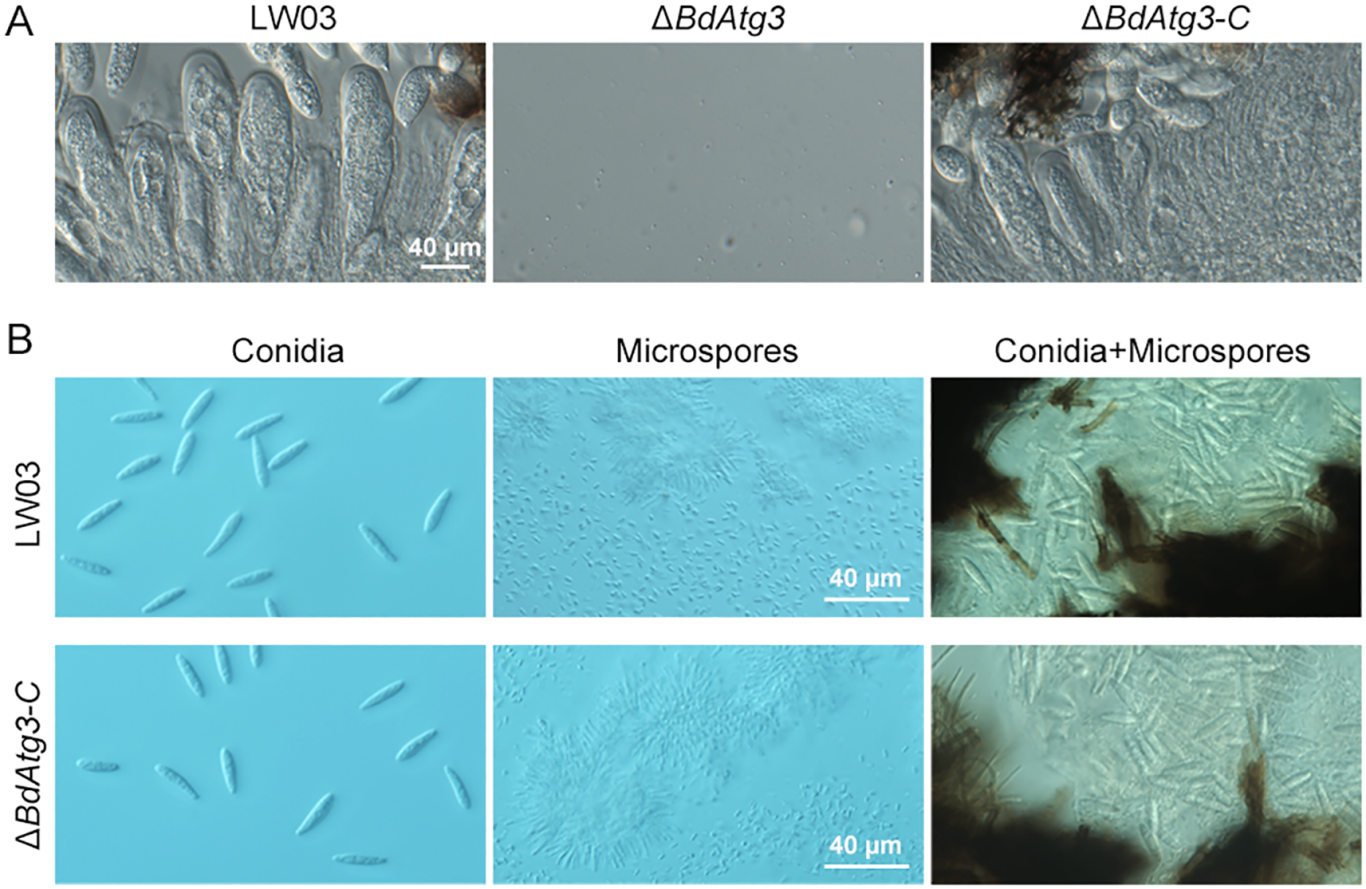
BdAtg3 is essential for sexual and asexual reproduction. **(A)** Ascus containing ascospores formed on apple branches by the wild-type strain LW03 and complemented strain Δ*BdAtg3-C*. **(B)** Conidia and microspores produced by each strain on young apple fruits.

Reproductive development, encompassing both sexual and asexual cycles, represents a critical biological process in ascomycetes that directly influences disease occurrence and epidemics (Liu et al., 2022b). To elucidate the functional role of BdAtg3 in the reproductive biology of *B. dothidea*, we systematically examined the sexual morphogenesis and conidiogenesis under microscope (Olympus CX43, Tokyo, Japan). As shown in **Figure 4A**, the Δ*BdAtg3* mutant was incapable of ascus formation after 30 days of incubation, in contrast to the prolific production of ascus containing viable ascospores by both the wild-type strain LW03 and complemented strain Δ*BdAtg3-C*. In addition, after 7 days of incubation with black light exposure at 25°C, LW03 and Δ*BdAtg3-C* produced abundant fruiting bodies containing both conidia and microspores on disease lesions, while the Δ*BdAtg3* mutant lost ability to produce fruiting body (**Figure 4B**). These results indicate that BdAtg3 is indispensable for both sexual and asexual reproductive processes in *B. dothidea*.

### 3.5 BdAtg3 is required for virulence

**Figure 5:**
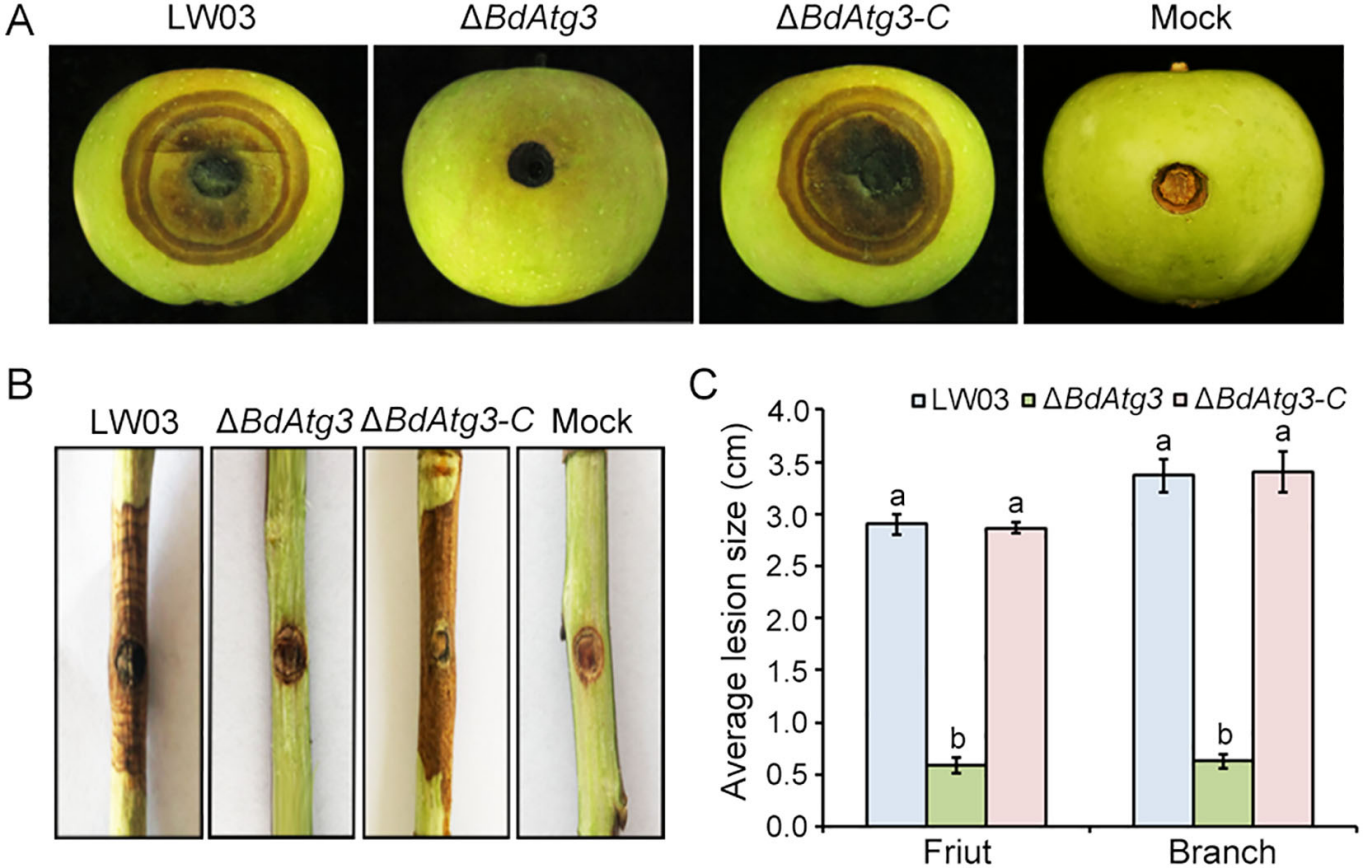
BdAtg3 is required for virulence. Disease symptoms on young apple fruits **(A)**, and branches **(B)**, caused by the wild-type strain LW03, *BdATG3* deletion mutant Δ*BdAtg3* and complemented strain Δ*BdAtg3-C*. **(C)** Lesion size on apple fruit and branches caused by each strain. Values on the bars followed by the same letter are not significantly different at *P* = 0.05.

To determine the contribution of BdAtg3 to the virulence of *B. dothidea*, we conducted pathogenicity assays using both young apple fruits and one-year-old apple branches. The wild-type strain LW03 and complemented strain Δ*BdAtg3-C* caused severe disease symptoms characterized by extensive tissue maceration and discoloration on apple fruits at 7 days post-inoculation. In contrast, the Δ*BdAtg3* mutant exhibited significantly reduced virulence, producing only minor necrotic lesions (**Figure 5A**). This attenuation in pathogenicity was consistently observed in branch infection assays, where Δ*BdAtg3* showed markedly decreased colonization efficiency compared to LW03 and Δ*BdAtg3-C* (**Figures 5B, C**). These results indicate that BdAtg3 plays an important role in the full virulence of *B. dothidea*.

### 3.6 BdAtg3 influences nuclei distribution

**Figure 6:**
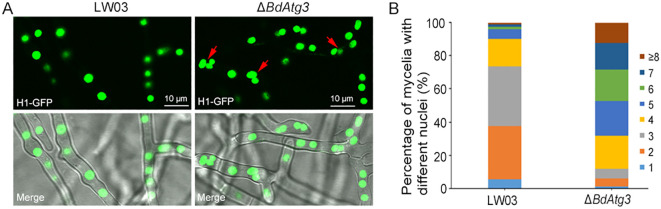
BdAtg3 influences nuclei distribution. **(A)** Nuclei distribution in mycelia of the wild-type strain LW03 and *BdATG3* deletion mutant Δ*BdAtg3*. **(B)** The proportion of nuclei in mycelial compartment of each strain.

Previous studies have demonstrated the involvement of autophagy in modulating nuclear dynamics in filamentous fungi (Corral-Ramos et al., 2015). To investigate the potential role of BdAtg3 in nuclear distribution of *B. dothidea*, the nuclei in mycelial compartments of each strain was checked through GFP labeled histone H1 observation (Liu et al., 2022b). As shown in **Figure 6**, the wild-type strain LW03 predominantly exhibited 2-4 nuclei per mycelial compartment, whereas the Δ*BdAtg3* mutant showed significantly increased nuclear numbers, typically ranging from 4-8 nuclei per compartment. These results suggest that BdAtg3 plays a crucial regulatory role in maintaining proper nuclear dynamics during hyphal development in *B. dothidea*.

## 4 Discussion

Autophagy is a vital catabolic process that maintains cellular homeostasis, ensuring the normal functioning of eukaryotic cells (Ryter et al., 2013). Recent research has highlighted the significant role of autophagy in the growth, development, and virulence of filamentous fungi (Khan et al., 2012; Pollack et al., 2009). In this study, we identified and characterized the E2-like activating enzyme Atg3 in *Botryosphaeria dothidea*. Phylogenetic tree analysis revealed that BdAtg3 is highly conserved in the Phylum Ascomycota, and shared closely cluster with the orthologs from *F. graminearum*, *M. oryzae*, *S. macrospora*, *N. crassa*, *C. gleosporiodes*, and *A. oryzae* (**Figure 1**). Moreover, the deletion of *BdATG3* completely inhibited the autophagic process in *B. dothidea* (**Figure 2**), which is consistent with the observations in *Saccharomyces cerevisiae* and other filamentous fungi (Khalid et al., 2019; Ngu et al., 2015).

Recycling of intracellular components through autophagy is essential for providing the necessary materials and energy to support fungal vegetative growth and development (Ren et al., 2017; Shoji et al., 2006). In this study, the Δ*BdAtg3* mutant showed a reduced mycelial growth rate on PDA plates and produced fewer aerial hyphae (**Figure 3**), which is consistent with the role of Atg3 in the growth of aerial hyphae in some filamentous fungi, such as *Botrytis cinerea* and *Fusarium graminearum* (Lv et al., 2017; Ren et al., 2018b). Additionally, the Δ*BdAtg3* mutant lost ability to produce ascospores and conidia under suitable conditions (**Figure 4**). These findings are line with the role of Atg3 in regulating both sexual and asexual reproduction in some other filamentous fungi (Pollack et al., 2009). The infection process of pathogenic fungi depends critically on autophagic degradation to fulfill the increased metabolic and energy demands during host invasion (Liu et al., 2012; Ren et al., 2018a). Consistent with findings in other fungal pathogens (Liu et al., 2016), the Δ*BdAtg3* mutant showed severely compromised infectivity in both apple fruits and branches (**Figure 5**), underscoring the crucial role of BdAtg3 in the virulence of *B. dothidea*.

Autophagy contributes to regulation of nuclear dynamics during vegetative growth and hyphal fusion (Corral-Ramos et al., 2015). In this study, the Δ*BdAtg3* mutant exhibited significantly increased nuclear numbers in mycelial compartment compared with the wild-type strain (**Figure 6**), suggesting the relatively conservative role of autophagy in regulating nuclear distribution.

In conclusion, our study demonstrated that BdAtg3 is essential for autophagy process, and significantly contributes to mycelial growth, development, and virulence in *B. dothidea*, which provides a theoretical basis for the development of novel targeted fungicide to control apple ring rot disease.

## Data Availability

The datasets presented in this study can be found in online repositories. The names of the repository/repositories and accession number(s) can be found in the article/**Supplementary Material**.

## Supplementary material

Supplementary Figure 1 Generation and identification of the *BdATG3* deletion mutant in *Botryosphaeria dothidea*. **(A)** Schematic diagram of the homologous recombination strategy for *BdATG3* replacement. **(B)** PCR identification of the Δ*BdAtg3* mutants. M represents DNA marker.

Supplementary Table 1 PCR primers used in this study.

## References

[B1] Corral-Ramos C. Roca M. G. Di Pietro A. Roncero M. I. Ruiz-Roldán C. (2015). Autophagy contributes to regulation of nuclear dynamics during vegetative growth and hyphal fusion in *Fusarium oxysporum* . Autophagy 11, 131–144. doi: 10.4161/15548627.2014.994413, PMID: 25560310 PMC4507430

[B2] Dong X. L. Cheng Z. Z. Leng W. F. Li B. H. Xu X. M. Lian S.. (2021). Progression of symptoms caused by *Botryosphaeria dothidea* on apple branches. Phytopathology 111, 1551–1559. doi: 10.1094/PHYTO-12-20-0551-R, PMID: 33487023

[B3] Eskelinen E. L. (2019). Autophagy: Supporting cellular and organismal homeostasis by self-eating. Int. J. Biochem. Cell Biol. 111, 1–10. doi: 10.1016/j.biocel.2019.03.010, PMID: 30940605

[B4] Fang D. Xie H. Hu T. Shan H. Li M. (2021). Binding features and functions of ATG3. Front. Cell Dev. Biol. 9, 685625. doi: 10.3389/fcell.2021.685625, PMID: 34235149 PMC8255673

[B5] Khalid A. R. Lv X. Naeem M. Mehmood K. Shaheen H. Dong P.. (2019). Autophagy related gene (ATG3) is a key regulator for cell growth, development, and virulence of *Fusarium oxysporum* . Genes 10, 658. doi: 10.3390/genes10090658, PMID: 31466418 PMC6769740

[B6] Khan I. A. Lu J. P. Liu X. H. Rehman A. Lin F. C. (2012). Multifunction of autophagy-related genes in filamentous fungi. Microbiol. Res. 167, 339–345. doi: 10.1016/j.micres.2012.01.004, PMID: 22554685

[B7] Liu X. H. Gao H. M. Xu F. Lu J. P. Devenish R. J. Lin F. C. (2012). Autophagy vitalizes the pathogenicity of pathogenic fungi. Autophagy 8, 1415–1425. doi: 10.4161/auto.21274, PMID: 22935638

[B8] Liu N. Lian S. Zhou S. Wang C. Ren W. Li B. (2022a). Involvement of the autophagy-related gene *BdATG8* in development and pathogenicity in *Botryosphaeria dothidea* . J. Integr. Agric. 21, 2319–2328. doi: 10.1016/S2095-3119(21)63863-7

[B9] Liu X. H. Xu F. Snyder J. H. Shi H. B. Lu J. P. Lin F. C. (2016). Autophagy in plant pathogenic fungi. Semin. Cell Dev. Biol. 57, 128–137. doi: 10.1016/j.semcdb.2016.03.022, PMID: 27072489

[B10] Liu N. Zhu M. Zhang Y. Wang Z. Li B. Ren W. (2022b). Involvement of the autophagy protein Atg1 in development and virulence in *Botryosphaeria dothidea* . J. Fungi. 8, 904. doi: 10.3390/jof8090904, PMID: 36135629 PMC9501979

[B11] Lv W. Wang C. Yang N. Que Y. Talbot N. J. Wang Z. (2017). Genome-wide functional analysis reveals that autophagy is necessary for growth, sporulation, deoxynivalenol production and virulence in *Fusarium graminearum* . Sci. Rep. 7, 11062. doi: 10.1038/s41598-017-11640-z, PMID: 28894236 PMC5594004

[B12] Marsberg A. Kemler M. Jami F. Nagel J. H. Postma-Smidt A. Naidoo S.. (2017). *Botryosphaeria dothidea*: a latent pathogen of global importance to woody plant health. Mol. Plant Pathol. 18, 477–488. doi: 10.1111/mpp.12495, PMID: 27682468 PMC6638292

[B13] Martens S. Fracchiolla D. (2020). Activation and targeting of ATG8 protein lipidation. Cell Discov. 6, 23. doi: 10.1038/s41421-020-0155-1, PMID: 32377373 PMC7198486

[B14] Mizushima N. (2007). Autophagy: process and function. Genes Dev. 21, 2861–2873. doi: 10.1101/gad.1599207, PMID: 18006683

[B15] Mizushima N. (2020). The ATG conjugation systems in autophagy. Curr. Opin. Cell Biol. 63, 1–10. doi: 10.1016/j.ceb.2019.12.001, PMID: 31901645

[B16] Nair U. Thumm M. Klionsky D. J. Krick R. (2011). GFP-Atg8 protease protection as a tool to monitor autophagosome biogenesis. Autophagy 7, 1546–1550. doi: 10.4161/auto.7.12.18424, PMID: 22108003 PMC3327617

[B17] Ngu M. Hirata E. Suzuki K. (2015). Visualization of Atg3 during autophagosome formation in *Saccharomyces cerevisiae* . J. Biol. Chem. 290, 8146–8153. doi: 10.1074/jbc.M114.626952, PMID: 25645919 PMC4375471

[B18] Ohsumi Y. (2012). Yoshinori Ohsumi: autophagy from beginning to end. Interview by Caitlin Sedwick. J. Cell Biol. 197, 164–165. doi: 10.1083/jcb.1972pi, PMID: 22508506 PMC3328387

[B19] Parzych K. R. Klionsky D. J. (2014). An overview of autophagy: morphology, mechanism, and regulation. Antioxid. Redox Signal 20, 460–473. doi: 10.1089/ars.2013.5371, PMID: 23725295 PMC3894687

[B20] Pollack J. K. Harris S. D. Marten M. R. (2009). Autophagy in filamentous fungi. Fungal Genet. Biol. 46, 1–8. doi: 10.1016/j.fgb.2008.10.010, PMID: 19010432

[B21] Ren W. Liu N. Sang C. Shi D. Zhou M. Chen C.. (2018a). The autophagy gene *BcATG8* regulates the vegetative differentiation and pathogenicity of *Botrytis cinerea* . Appl. Environ. Microbiol. 84, e02455–e02417. doi: 10.1128/AEM.02455-17, PMID: 29572212 PMC5960959

[B22] Ren W. Sang C. Shi D. Song X. Zhou M. Chen C. (2018b). Ubiquitin-like activating enzymes BcAtg3 and BcAtg7 participate in development and pathogenesis of *Botrytis cinerea* . Curr. Genet. 64, 919–930. doi: 10.1007/s00294-018-0810-3, PMID: 29417220

[B23] Ren W. Zhang Z. Shao W. Yang Y. Zhou M. Chen C. (2017). The autophagy-related gene *BcATG1* is involved in fungal development and pathogenesis in *Botrytis cinerea* . Mol. Plant Pathol. 18, 238–248. doi: 10.1111/mpp.12396, PMID: 26972592 PMC6638273

[B24] Ren W. Zhang Y. Zhu M. Liu Z. Lian S. Wang C.. (2023). The phosphatase cascade Nem1/Spo7-Pah1 regulates fungal development, lipid homeostasis, and virulence in *Botryosphaeria dothidea* . Microbiol. Spectr. 11, e0388122. doi: 10.1128/spectrum.03881-22, PMID: 37191532 PMC10269782

[B25] Ryter S. W. Cloonan S. M. Choi A. M. (2013). Autophagy: a critical regulator of cellular metabolism and homeostasis. Mol. Cells 36, 7–16. doi: 10.1007/s10059-013-0140-8, PMID: 23708729 PMC3887921

[B26] Shoji J. Y. Arioka M. Kitamoto K. (2006). Possible involvement of pleiomorphic vacuolar networks in nutrient recycling in filamentous fungi. Autophagy 2, 226–227. doi: 10.4161/auto.2695, PMID: 16874107

[B27] Sun Z. Hao B. Wang C. Li S. Xu Y. Li B.. (2023). Biocontrol features of *Pseudomonas syringae* B-1 against *Botryosphaeria dothidea* in apple fruit. Front. Microbiol. 14, 1131737. doi: 10.3389/fmicb.2023.1131737, PMID: 36937290 PMC10017730

[B28] Wang L. Tu H. Hou H. Zhou Z. Yuan H. Luo C.. (2022). Occurrence and detection of carbendazim resistance in *Botryosphaeria dothidea* from apple orchards in China. Plant Dis. 106, 207–214. doi: 10.1094/PDIS-06-20-1204-RE, PMID: 34227835

[B29] Yin Z. Chen C. Yang J. Feng W. Liu X. Zuo R.. (2019). Histone acetyltransferase MoHat1 acetylates autophagy-related proteins MoAtg3 and MoAtg9 to orchestrate functional appressorium formation and pathogenicity in *Magnaporthe oryzae* . Autophagy 15, 1234–1257. doi: 10.1080/15548627.2019.1580104, PMID: 30776962 PMC6613890

[B30] Yu J. H. Hamari Z. Han K. H. Seo J. A. Reyes-Domínguez Y. Scazzocchio C. (2004). Double-joint PCR: a PCR-based molecular tool for gene manipulations in filamentous fungi. Fungal Genet. Biol. 41, 973–981. doi: 10.1016/j.fgb.2004.08.001, PMID: 15465386

[B31] Zhao Y. G. Zhang H. (2018). Formation and maturation of autophagosomes in higher eukaryotes: a social network. Curr. Opin. Cell Biol. 53, 29–36. doi: 10.1016/j.ceb.2018.04.003, PMID: 29727743

[B32] Zhu M. Huan T. Ma Y. Han Y. Liu N. Lian S.. (2024). The two-component histidine kinase BdHk1 regulates fungal development, virulence and fungicide sensitivity in *Botryosphaeria dothidea* . Pest. Biochem. Physiol. 205, 106134. doi: 10.1016/j.pestbp.2024.106134, PMID: 39477586

