# Supplementary figures and images for: Involvement of the E2-like enzyme Atg3 in fungal development and virulence of Botryosphaeria dothidea
Source: Front Plant Sci. 2025 Aug 15;16:1590359. doi: 10.3389/fpls.2025.1590359 (PMC12395508; doi:10.3389/fpls.2025.1590359)

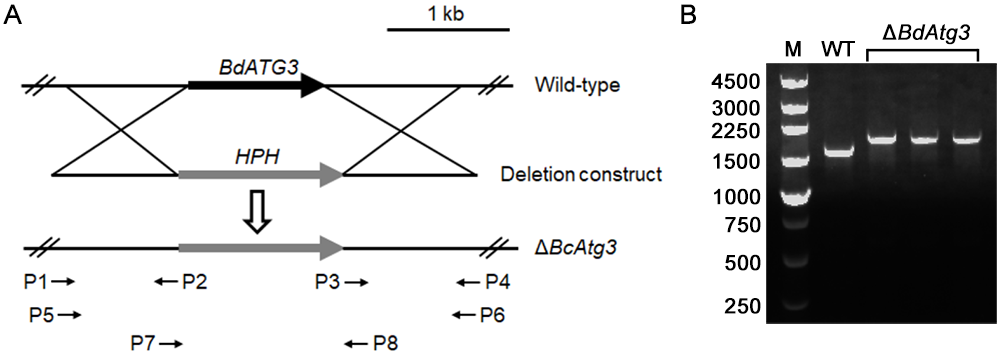

Supplement: Supplementary Figure 1 — Generation and identification of the BdATG3 deletion mutant in Botryosphaeria dothidea. (A) Schematic diagram of the homologous recombination strategy for BdATG3 replacement. (B) PCR identification of the ΔBdAtg3 mutants. M represents DNA marker. [file Image1.tif]
