# Supplementary material for: Involvement of the E2-like enzyme Atg3 in fungal development and virulence of Botryosphaeria dothidea
Source: Front Plant Sci. 2025 Aug 15;16:1590359. doi: 10.3389/fpls.2025.1590359 (PMC12395508; doi:10.3389/fpls.2025.1590359)
Supplement: Supplementary Table 1 — PCR primers used in this study. [file Table1.docx]

**Table S1.** PCR primers used in this study

| Primer | Sequence (5’-3’) | Relevant characteristics |
| --- | --- | --- |
| P1  P2 | GCTCTATGATCTGGGCCGCG  CAAAATAGGCATTGATGTGTTGACCTCCCTGTTGTGGGGCGCTTCGAG | PCR primers to amplify *BdATG3* upstream fragment for construction of the *BdATG3* deletion fragment |
| P3  P4 | CTCGTCCGAGGGCAAAGGAATAGAGTAGGAAGGAGATTCCCATATAAC  CTCGTGACGAGCTTCTCGTC | PCR primers to amplify *BdATG3* downstream fragment for construction of the *BdATG3* deletion fragment |
| P5  P6 | CGAATTGCTCACGGAGGTCC  CTTCGAGAGATCAAGAATCC | PCR primers to amplify the *BdATG3* deletion fragment for generation of the *BdATG3* deletion mutants |
| P7  P8 | CAGCTACTGACTGGTACGTC  GGTGGTATATCTGTGTGTAG | PCR primers for identification of the *BdATG3* deletion mutants |
| HPH-F  HPH-R | GGAGGTCAACACATCAATGC  CTACTCTATTCCTTTGCCCT | PCR primers to amplify the hygromycin resistance gene (*HPH*) |
| ATG3-GFP-F  ATG3-GFP-R | ACTCACTATAGGGCGAATTGGGTACTCAAATTGGTTGATTTGCAGGAGGAAGGTCA  CACCACCCCGGTGAACAGCTCCTCGCCCTTGCTCACTACACCCATAGTGAAGTCGT | PCR primers to amplify the native promoter and open reading frame of *BdATG3* to construct BdATG3-GFP fusion vector |
| ATG3-ID-F  GFP-ID-R | CAGGACAAGATGTTAGCAAG  CGTGCTGCTTCATGTGGTCG | PCR primers for identification of the PYF11-BdATG3-GFP vector |
